# Efficacy of CytoSorb®: a systematic review and meta-analysis

**DOI:** 10.1186/s13054-023-04492-9

**Published:** 2023-05-31

**Authors:** Sören Becker, Hannah Lang, Clara Vollmer Barbosa, Zhejia Tian, Anette Melk, Bernhard M. W. Schmidt

**Affiliations:** 1 Department of Nephrology and Hypertension, Hannover Medical School, Carl-Neuberg-Straße 1, 30625 Hannover, Germany; 2 Department of Pediatric Kidney, Liver and Metabolic Diseases, Hannover Medical School, Hannover, Germany

## Abstract

**Introduction:**

Cytokine adsorption using the CytoSorb® adsorber has been proposed in various clinical settings including sepsis, ARDS, hyperinflammatory syndromes, cardiac surgery or recovery after cardiac arrest. The aim of this analysis is to provide evidence for the efficacy of the CytoSorb® adsorber with regard to mortality in various settings.

**Methods:**

We searched PubMed, Cochrane Library database and the database provided by Cytosorbents™ (01.1.2010–29.5.2022). We considered randomized controlled trials and observational studies with control groups. The longest reported mortality was defined as the primary endpoint. We computed risk ratios and 95%-confidence intervals and used DerSimonian and Lairds random effects model. We analysed all studies combined and divided them into the subgroups: sepsis, cardiopulmonary bypass surgery (CPB), other severe illness, SARS-CoV-2 infection and recovery from cardiac arrest. The meta-analysis was registered in advance (PROSPERO: CRD42022290334).

**Results:**

Of an initial 1295 publications, 34 studies were found eligible, including 1297 patients treated with CytoSorb® and 1314 controls. Cytosorb® intervention did not lower mortality (RR [95%-CI]: all studies 1.07 [0.88; 1.31], sepsis 0.98 [0.74; 1.31], CPB surgery 0.91 [0.64; 1.29], severe illness 0.95 [0.59; 1.55], SARS-CoV-2 1.58 [0.50; 4.94]). In patients with cardiac arrest, we found a significant survival advantage of the untreated controls (1.22 [1.02; 1.46]). We did not find significant differences in ICU length of stay, lactate levels, or IL-6 levels after treatment. Of the eligible 34 studies only 12 were randomized controlled trials. All observational studies showed moderate to serious risk of bias.

**Interpretation:**

To date, there is no evidence for a positive effect of the CytoSorb® adsorber on mortality across a variety of diagnoses that justifies its widespread use in intensive care medicine.

**Supplementary Information:**

The online version contains supplementary material available at 10.1186/s13054-023-04492-9.

## Introduction

Massive release of cytokines into the bloodstream is the pathophysiological culprit of many life-threating diseases. Pro-inflammatory cytokines lead to vasodilation, capillary leakage, and coagulopathy. Anti-inflammatory cytokines can cause relative immunosuppression leading to secondary nosocomial infections. The uncontrolled release of both types of cytokines has the potential to end in multiple organ failure [1].

Various blood purification techniques, such as dialysis using high cut-off membranes, hemoadsorption, high volume hemofiltration, and plasma exchange have been proposed to unselectively reduce cytokine levels [2]. CytoSorb® is one of the most widely used blood purification devices, which can reduce the level of hydrophobic molecules with a molecular mass up to 55 kDa. [3] Thus, the device adsorbs cytokines, bile acids, and myoglobin. CytoSorb® is in clinical use in patients with an excessive immune response such as in sepsis, ARDS, SARS-CoV-2 infections, hyperinflammatory syndromes, and during and after cardiac surgery using cardiopulmonary bypass (CPB). In addition, CytoSorb® may be useful in liver failure, elimination of DOACs or certain acute intoxications [4]. The device is considered to be safe and well-tolerated. However, there is no consensus on the effectiveness.

The aim of this systematic review and meta-analysis is to evaluate the impact of CytoSorb® in all previously described medical conditions. The primary endpoint is the longest reported mortality. Furthermore, ICU length of stay, norepinephrine requirements, IL-6 and lactate levels will be compared.

## Methods

We performed a systematic search of the PubMed and Cochrane Library database. We used “CytoSorb” as the keyword in all fields. Related articles were evaluated for additional publications. In addition, the database provided by CytoSorbents (https://literature.cytosorb-therapy.com/?_ga=2.58770730.642024760.1646225438-27175153.1645687922) was screened. The last research update was conducted on May 29 2022.

We considered randomized controlled trials (RCT) and observational studies comprising a control group. Case reports were excluded. Studies had to contain information on the primary endpoint. If the results of multiple studies referred to the same patients, the larger study was considered. The intervention group had to receive at least one treatment with the CytoSorb® adsorber. The control group was allowed to differ only by the CytoSorb® treatment. Intervention and control groups each had to include at least three patients. There was no language restriction. Studies were selected by two investigators independently; in case of disagreement, consensus was sought. In accordance with the recommendations of the Bias Method Group of the Cochrane network, the quality of the studies was assessed using the ROBINS-I tool (Risk of Bias in Non-randomized Studies of Interventions) or the RoB 2 tool (revised tool for Risk of Bias in randomized trials). The data were collected by two investigators independently and summarized in an Excel file. If graphs were provided instead of exact values, we analysed the data using “WebPlotDigitizer” (https://apps.automeris.io/wpd/).

The primary endpoint was the longest reported mortality (30-day, in-hospital or ICU mortality). If more than one value was given, the value with the longest observational period was chosen. The primary endpoint was computed as relative risk. Subgroup analyses of the primary endpoint were performed for different medical conditions (sepsis, CPB surgery, severe illness, SARS-CoV-2 and cardiac arrest). Subgroup analyses for different study designs were also performed (randomized controlled trials vs. observational studies with and without propensity score matched controls). The random effect model (DerSimonian and Laird) was used for inference.

Secondary endpoints were ICU length of stay and hospital length of stay. We extracted information on norepinephrine dose, mean arterial pressure (MAP), CRP, PCT, lactate and interleukin-6 (IL-6) levels before and after CytoSorb® intervention. The levels of inflammatory markers after CytoSorb® intervention were defined as the first reported value after the start of CytoSorb® treatment. In addition, we evaluated the SOFA, SAPS-2, and APACHE-2 scores to compare the control and intervention groups. Also, we screened all studies for reported adverse events. Secondary endpoints were reported as risk differences (treatment group—control group) for all studies combined and for the individual subgroups. Analyses of secondary end points were performed if at least two studies reported relevant data. For elective cardiopulmonary bypass surgery, sepsis scores and physiologic endpoints were not applicable. The original studies reported secondary endpoints most often as mean or median values. We followed the recommendations of McGrath et al [5]: assuming normal distribution, mean values were considered to be equal to medians. Medians reported with interquartile ranges or minimum and maximum values were converted to mean values and standard deviation using the method of Luo et al. [6] Subsequently, the effect of mean values was assessed using a random effect model with the DerSimonian-Laird method. Effects of median values were calculated by the quantile estimation method. Information on secondary endpoints is subsequently always reported first as mean and then as median.

Trial sequential analysis (TSA) was performed using the software provided by the Copenhagen Trial Unit on https://ctu.dk. We performed TSA of RCTs in the subgroup of CPB only, as this subgroup had the highest number of studies. We used the following settings for the analysis: RRR to detect 10%, Power 20%, *p* < 0.05 two-sided, *α*-spending function: O’Brien Flemming; the mortality in the control group was 14.85 [7].

Risk of bias was assessed with the tools ROBINS-I and RoB 2 by two investigators independently. We used funnel plots to assess publication bias and applied the GRADE methodology to assess certainty. We used R 4.1.0 with the packages meta, metafor, metamedian, lqmm, hmisc, estmeansd, forestplot and writexl for all analyses. *P* values less than 0.05 were considered to be statistically significant. Heterogeneity was assessed using *I*^2^ and Cochrane’s *q*. The study protocol was registered on the PROSPERO database (registration number: CRD42022290334).

## Results

### Study search

**Fig. 1:**
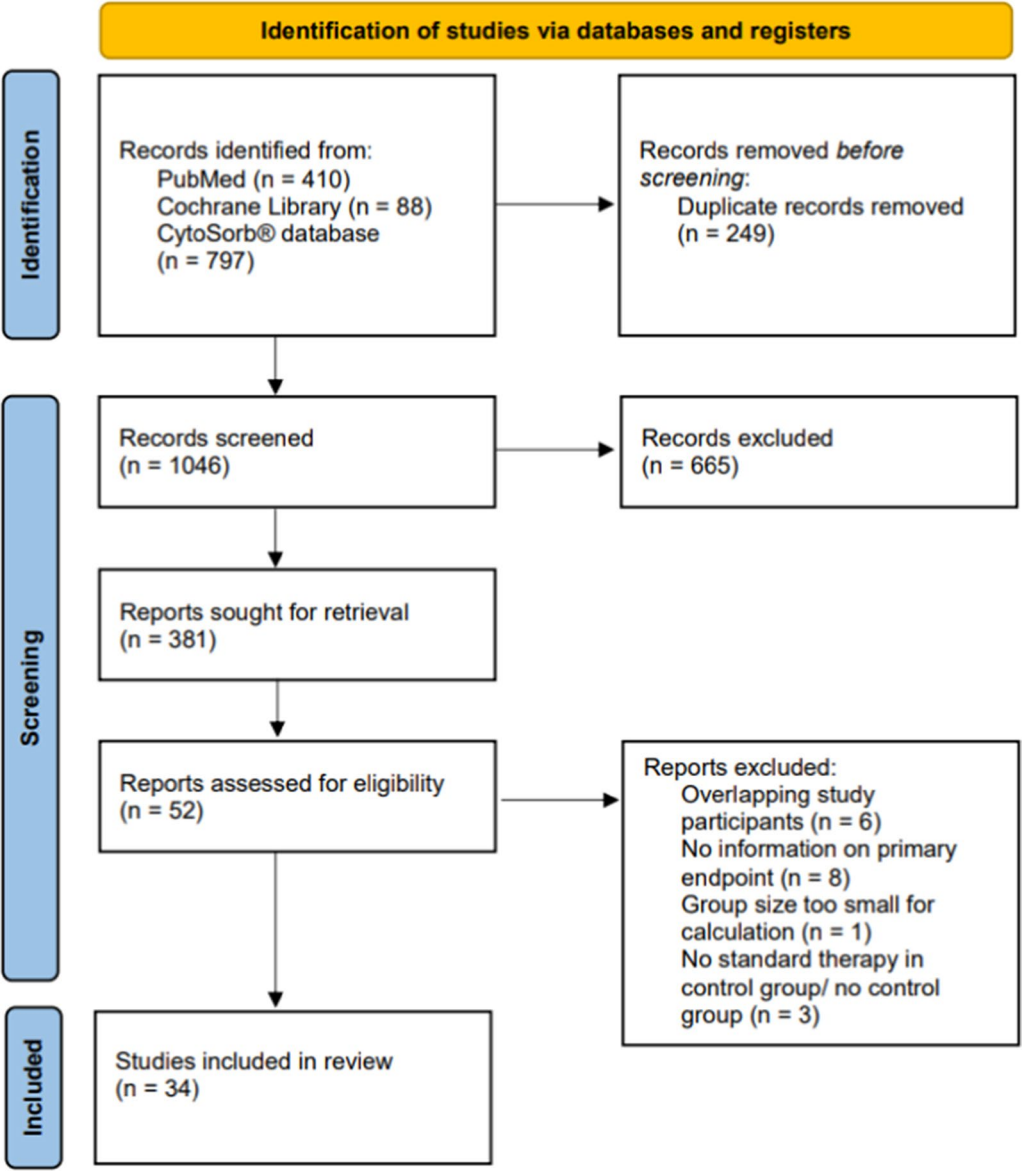
Flow diagram

The search returned 1295 hits, of which 249 were excluded due to duplication. One thousand and forty-six titles were screened. Of 381 reports, first the abstracts and then the full texts were reviewed for eligibility. Six studies were excluded due to overlapping study participants [8–13]. Another eight studies did not provide information on our defined primary endpoint [14–21]. One study was excluded because the group size was too small (*n* = 2), and three studies were excluded due to the lack of standard therapy in the control group [3, 22–24]. Thus, 34 studies were available for analysis (Fig. 1). No paediatric patients were included since all studies involving children were either case reports or did not have a control group.

### Study characteristics

Eight studies investigated the effect of CytoSorb® in patients with sepsis or septic shock [25–32]. Fifteen studies used it in cardiopulmonary bypass surgery [33–47]. Four studies employed CytoSorb® in patients suffering from SARS-CoV-2 [48–51]. Another three studies used CytoSorb® in patients after cardiovascular arrest [52–54]. There were four other studies that investigated the effect of CytoSorb® yet could not be assigned to any of the previously mentioned groups. We defined this group as “severe illness” and included the following conditions: severely injured polytrauma patients, patients with ARDS requiring veno-venous-ECMO, severely ill patients with an IL-6 ≥ 10,000 pg/ml and patients with severe acute pancreatitis [55–58]. There were twelve randomized controlled trials (RCT), twelve observational studies in which intervention and control groups were matched by propensity scores (PSM), and ten observational studies with otherwise selected controls (nPSM) (Table 1). A total of 1297 patients were treated with CytoSorb® and compared to 1314 patients without CytoSorb®.

**Table 1:** Study characteristics

First author	Year	Study design	Patient characteristics	Treatment scheme	N (CytoSorb®/Control)	Age (CytoSorb®/Control) M or Md	Sex (% male) (CytoSorb®/Control)
Schädler	2017	RCT, open-label, multi-centre	Severe sepsis or septic shock and ARDS	6 h per day for up to 7 consecutive days	47/50	66/65	74/70
Hawchar	2019	RCT, open-label, single-centre	Early (< 24 h) onset of septic shock, mechanical ventilation, norepinephrine > 10 μg/min	1 treatment for 24 h	10/10	60/71	70/60
Brouwer	2019	Retrospective PS weighted register study, single-centre	Septic shock with CRRT treated on ICU	Treatment until improvement	67/49	61/69	55/61
Akil	2020	Retrospective control group, prospective intervention group, single-centre	Pneumogenic sepsis and ECMO therapy	≥ 2 treatments; device changed every 24 h	13/7	61/61	38/29
Schittek	2020	Retrospective control group, prospective intervention group, single-centre	Septic shock with acute kidney injury and noradrenaline dose (> 20 µg/min)	≥ 1 treatment	43/33	63/62	88/72
Rugg	2020	Retrospective PS matched study, single-centre	Septic shock patients with RRT	≥ 1 treatment	42/42	64/68	64/60
Kogelmann	2021	Retrospective register study, multi-centre	Septic shock patients treated on ICU	≥ 1 treatment	198/69	62/66	61/NA
Garcia	2021	Retrospective control group, prospective intervention group, PS matched study, single-centre	severe, refractory septic shock	3 treatments for 24 h	48/48	57/58	65/65
Bernardi	2016	RCT, blinded, single-centre	Elective CPB surgery	Treatment during CPB	19/18	64/69	63/78
Träger	2017	Retrospective register study, single-centre	CPB surgery due to acute infective endocarditis	Treatment during CPB	39/28	61/72	69/71
Nemeth	2018	Prospective PS matched study, open-label, single-centre	Orthotopic heart transplantations without early postoperative death (72 h)	Treatment during CPB	16/16	51/50	88/78
Poli	2019	RCT, double-blinded, single-centre	Elective cardiac surgery with expected long CPB duration (> 120 min)	Treatment during CPB	15/15	67/69	88/73
Gleason	2019	RCT, open-label, multi-centre	Complex cardiac surgery with expected CPB duration > 3 h	2 parallel devices during CPB	23/23	66/61	56/78
Hassan	2019	Retrospective register study, single-centre	Emergency cardiac surgery under ticagrelor or rivaroxaban	Treatment during CPB	39/16	NA/ NA	66/81
Saller	2019	Retrospective PS matched study, single-centre	Aortic surgery with hypothermic circulatory arrest	Treatment during CPB	168/168	64/63	67/67
Wagner	2019	RCT, blinded, single-centre	Ross or David surgery	Treatment during CPB	13/10	50/54	87/100
Stupica	2020	RCT, double-blinded, single-centre	Elective high risk cardiac surgery with CPB duration > 90 min	Treatment during CPB	20/20	71/71	70/70
Haidari	2020	Retrospective register study, single-centre	Surgery of native mitral valve endocarditis	Treatment during CPB	30/28	59/61	70/57
Santer	2021	Retrospective PS weighted register study, single-centre	Valve surgery due to endocarditis	Treatment during CPB	41/200	66/65	92/78
Zhigalov	2021	Retrospective PS matched study, single-centre	LVAD implantation	Treatment during CPB	72/40	56/58	85/77
Asch	2021	RCT, open-label, single-centre	Cardiac surgery due to infective endocarditis	Treatment during CPB and 3 afterwards for 8 h each	10/10	65/69	70/90
Hassan	2022	Retrospective register study, single-centre	Emergency surgery for acute type A dissection under rivaroxaban or ticagrelor	treatment during CPB	10/11	75/62	40/55
Diab	2022	RCT, open-label, multi-centre	Surgery due to infective endocarditis	Treatment during CPB	138/144	69/69	71/77
Wilhelmi	2018	Retrospective register study, single-centre	Polytrauma	≥ 1 treatment for up to 4 days	5/5	35/32	100/100
Rieder	2021	Retrospective PS matched study, single-centre	ARDS treated with V-V ECMO and PCT > 3 ng/ml, IL-6 > 600 pg/ml	≥ 3 treatments	9/9	43/44	78/67
Scharf	2021	Retrospective PS matched study, single-centre	Serve illness (IL-6 > 10,000 pg/ml)	Treatment for at least 90 min	19/19	56/61	74/56
Rasch	2022	Retrospective control group, prospective intervention group, PS matched study, multi-centre	Acute pancreatitis, APACHE-II score of ≥ 10 and ≥ 1 marker of poor prognosis	2 treatments for 24 h each	16/32	52/60	75/66
Rampino	2020	Retrospective register study, single-centre	COVID-19, PaO2/FiO2 < 200 mmHg, CRP > 10 mg/dL, lymphocyte < 1500/mmc	2 treatments for 4 h each on 2 consecutive days	5/4	58/66	100/75
Schroeder	2020	Retrospective register study, single-centre	COVID-19 on ICU	≥ 1 treatment	13/57	NA/NA	NA/NA
Supady	2021	RCT, open-label, single-centre	COVID-19 pneumonia requiring ECMO	3 treatments for 24 h each	17/17	62/59	71/76
Stockmann	2022	RCT, open-label, single-centre	COVID-19 with vasoplegic shock (norepinephrine > 0.2 μg/kg/min, CRP 100 mg/L, and RRT)	≥ 3 treatments for 24 h each	23/26	61/66	91/77
Akin	2020	Prospective PS matched study, single-centre	Out-of-hospital cardiac arrest with increased vasopressor need	≥ 1 treatment for up to 3 days	24/48	62/61	83/83
Supady	2022 (a)	Retrospective PS matched study, single-centre	After cardiac arrest on ECMO	3 treatments for 24 h each	23/23	53/53	78/74
Supady	2022 (b)	RCT, open-label, single-centre	After cardiac arrest on ECMO	3 treatments for 24 h each	22/19	61/64	68/63

### Mortality

**Fig. 2:**
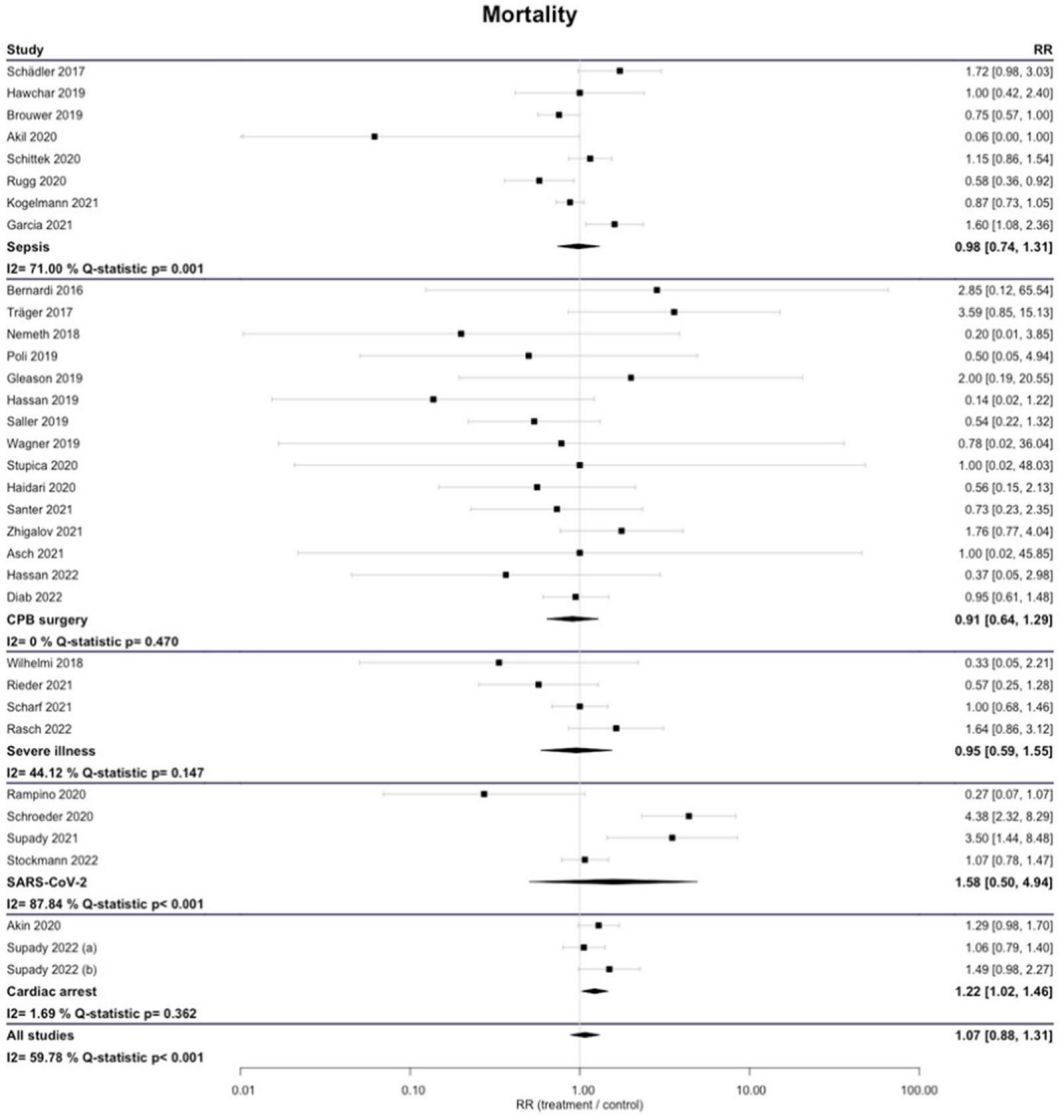
Longest-reported mortality by different diagnoses. *CPB* cardiopulmonary bypass surgery

The longest reported mortality was not significantly different between Cytosorb® and control groups for all studies combined (RR 1.07 [0.88; 1.31]) and in the subgroups sepsis (RR 0.98 [0.74; 1.31]), CPB surgery (RR 0.91 [0.64; 1.29]), severe illness (RR 0.95 [0.59; 1.55]), and SARS-CoV-2 (RR 1.58 [0.50; 4.94]). In patients with cardiac arrest, we found a significant survival advantage of the untreated controls (RR 1.22 [1.02; 1.46]) (Fig. 2). The results were very similar if ICU mortality, in-hospital mortality or 30-day mortality were assessed (Additional file 1: Figures S1–S3).

**Fig. 3:**
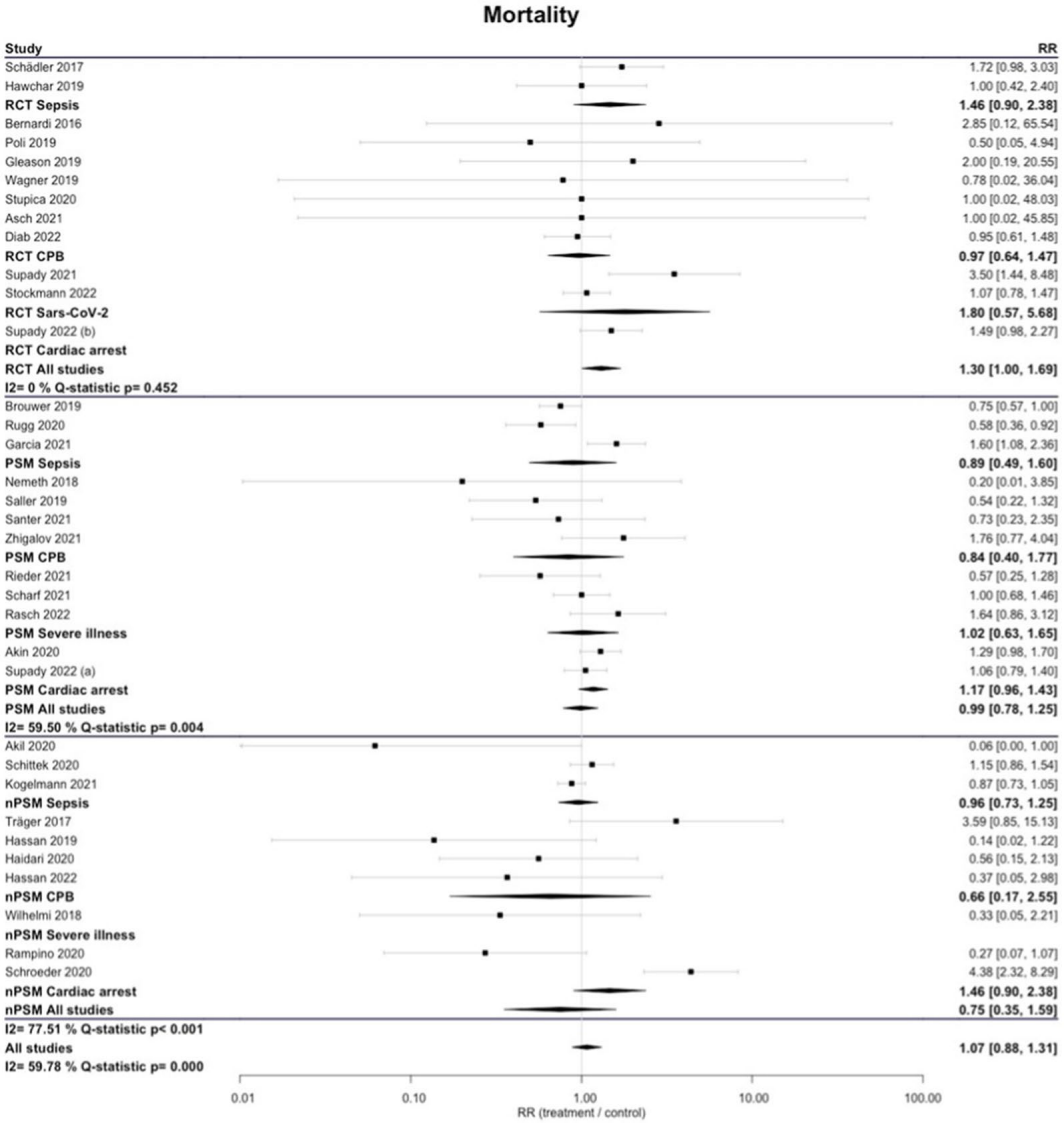
Longest-reported mortality by study design. *RCT* randomized controlled trial; *PSM* observational studies with propensity score matching; *nPSM* observational studies with matching other than propensity score matching

Subgroup analysis by study design showed that in none of the subgroups irrespective of the disease state examined and the quality of the study a significant difference in survival could be established. Comparing the pooled analysis across all disease states, one could get the impression the results get worse with higher quality of study design. (Fig. 3).

In some subgroups, we found high levels of heterogeneity. RCTs had significantly less between-study variance (I2 = 0%; *Q*-statistic *p* = 0.452) than observational studies (PSM I2 = 59.5%; Q-statistic *p* = 0.004; nPSM I2 = 77.51%; *Q*-statistic *p* < 0.001) (Figs. 2, 3).

### Trail sequential analysis

**Fig. 4:**
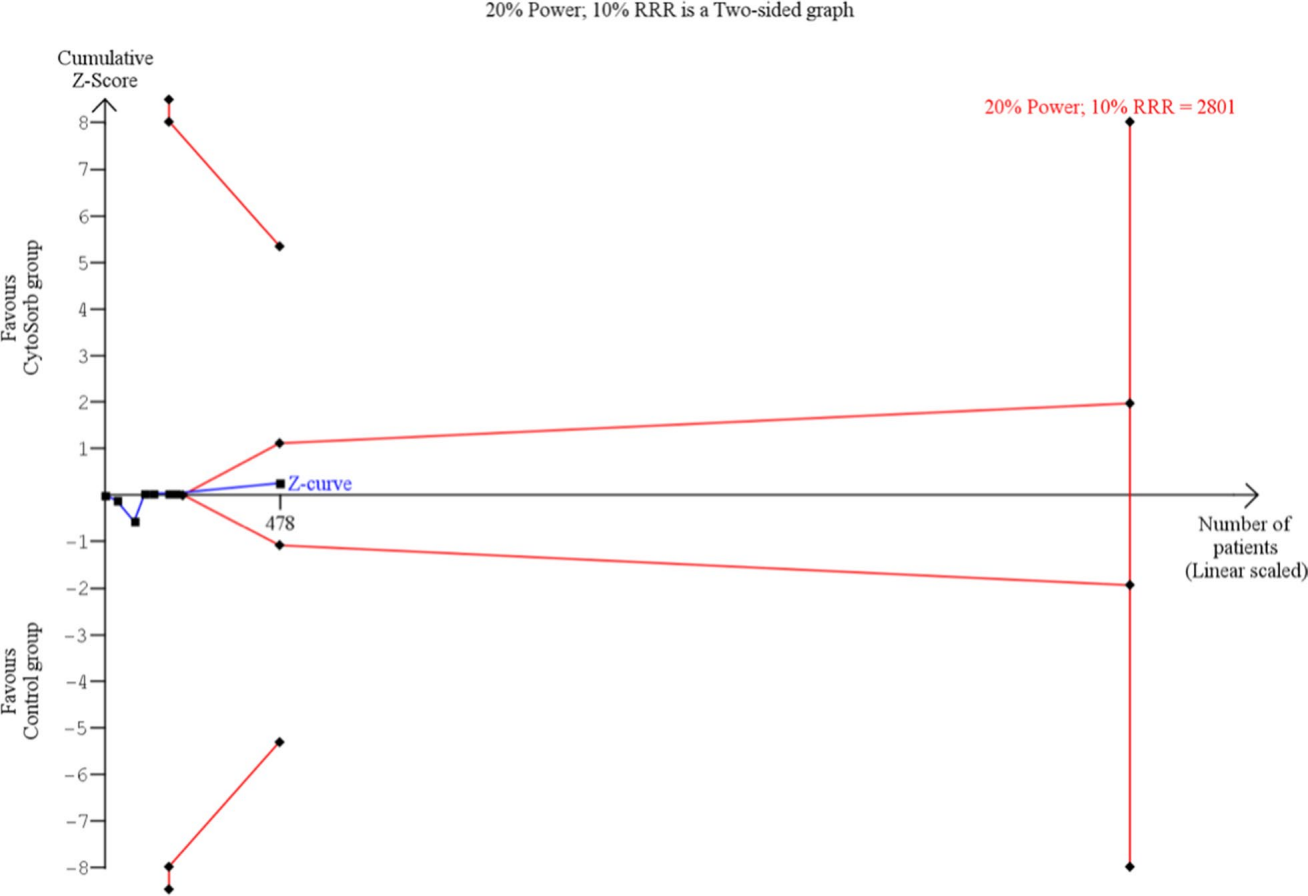
TSA of longest reported mortality after CPB

TSA was performed in the subgroup of CBP only for the primary endpoint. Figure 4 shows that with actual 478 patients the necessary number of 2801 is not reached. However, the cumulative Z-Score is already in the area of futility, suggesting that additional studies will not change the result.

### ICU and hospital length of stay

The ICU length of stay was not significantly different between CytoSorb® and control groups, neither for all studies combined, nor for subgroups of different diagnoses. The differences between groups (treatment group—control group) were compared first as mean values (± SD) and second as median values [and IQR] (Table 2).

**Table 2:** Hospital and ICU length of stay (difference in days: treatment group—control group)

	Hospital length of stay		ICU length of stay	
	M ± SD	Md [IQR]	M ± SD	Md [IQR]
Sepsis	3.43 (± 10.94)	0.51 [− 6.19; 7.21]	− 0.99 (± 3.32)	− 0.50 [− 3.19; 2.19]
CPB surgery	− 1.10 (± 2.89)	− 1.10 [− 4.06; 1.86]	0.38 (± 1.26)	0.27 [− 1.01; 1.55]
Severe illness				
SARS-CoV-2				
Cardiac arrest				
All studies	− 0.71 (± 2.75)	− 0.87 [− 3.64; 1.91]	0.29 (± 1.13)	0.30 [− 0.84; 1.44]

No statistically significant differences occurred

### Norepinephrine and mean arterial pressure (MAP)

There was no significant difference in norepinephrine dose (ug/kg/min) or MAP at baseline. For patients after cardiac arrest, the control group required a significantly higher norepinephrine dose when comparing mean values (− 0.16 (± 0.15)). This observation could not be confirmed when comparing medians (− 0.09 (± 0.26)). All other subgroups showed no significant difference between CytoSorb® and control groups in norepinephrine dose after the start of treatment. Furthermore, the blood pressure (MAP) 24 h after the start of treatment showed no significant difference (Table 3).

**Table 3:** Norepinephrine dose before and first reported after CytoSorb® treatment; MAP levels before and 24 h after CytoSorb® treatment (difference: treatment group—control group)

	Norepinephrine (µg/kg/min) before treatment		Norepinephrine (µg/kg/min) after treatment		MAP (mmHg) before treatment		MAP (mmHg) 24 h after treatment	
	M ± SD	Md [IQR]	M ± SD	Md [IQR]	M ± SD	Md [IQR]	M ± SD	Md [IQR]
Sepsis	0.03 (± 0.25)	0.05 [− 0.21; 0.31]	0.00 (± 0.15)	0.02 [− 0.11; 0.14]	2.07 (± 9.31)	1.81 [− 7.18; 10.80]		
CPB surgery			0.01 (± 0.01)	0.01 [− 0.01; 0.02]			1.64 (± 2.79)	2.44 [− 0.61; 5.49]
Severe illness								
SARS-CoV-2	− 0.03 (± 0.07)	− 0.02 [− 0.06; 0.02]	− 0.01 (± 0.04)	− 0.04 [− 0.10; 0.03]				
Cardiac arrest			**− 0.16 (± 0.15)**	− 0.09 [− 0.35; 0.18]				
All studies	− 0.02 (± 0.06)	− 0.02 [− 0.08; 0.04]	0.00 (± 0.02)	− 0.01 [− 0.02; 0.01]	1.51 (± 5.61)	1.12 [− 4.73; 6.97]	1.17 (± 2.65)	1.80 [− 1.07; 4.66]

Only the mean difference shown in bold was significant at the 0.05 level. Otherwise no statistically significant differences occurred

### Baseline SOFA, SAPS-2, and APACHE-2

None of these scores showed significant differences between CytoSorb® and control groups at baseline (Additional file 1: Table S1).

### CRP, PCT, lactate and IL-6 levels

The CRP levels were not significantly different at baseline or at first measurement after treatment (Additional file 1: Table S2).

PCT levels were significantly higher in the control group for patients with sepsis when considering median values (– 7.60 (± 7.76) / – 7.13 [– 13.29; – 0.97]). PCT levels in all studies combined and in patients with cardiac arrest were not significantly different. Furthermore, there were no significant differences in PCT levels after treatment (Additional file 1: Table S2).

There were no significant differences in lactate levels at baseline or at first measurement after treatment begin for any subgroup (Additional file 1: Table S3).

Solely the subgroup “severe disease” had significantly higher median IL-6 levels in the intervention group, the mean values showed no significant difference. After treatment there was also no difference in IL-6 levels between CytoSorb® and control groups in any subgroup (Additional file 1: Table S3).

### Adverse events

The occurrence of adverse events was monitored in 22 of the 34 studies. However, most studies did not clearly define what was considered an adverse event. Schädler et al. and Gleason et al. reported a decline in platelet count that they saw in connection with the CytoSorb® absorber [32, 40]. Other studies also observed a decline in platelet count that was found to be unrelated to the specific use of the CytoSorb® adsorber and more likely due to contact with an extracorporeal membrane in general [35, 36, 47, 52, 54]. None of the studies concluded that CytoSorb® is hazardous.

### Risk of bias

The risk of bias was assessed with the tools ROBINS-I and RoB 2 and publication bias by funnel plots. We applied the GRADE methodology to assess certainty. Some studies showed a considerable risk of bias (Additional file 1: Figs. S4 and S5). Using the ROBINS-I tool, all nPSM studies were rated as "serious risk”, since they did not measure or control for confounding factors. In addition, the funnel plot shows evidence of publication bias in nPSM studies with studies showing negative results remaining unpublished (Additional file 1: Fig. 6). In contrast, the PSM studies did control for confounding, so that most PSM studies were rated "moderate risk". However, some studies excluded certain confounders that we considered relevant (Nemeth et al. 2018 excluded the three significant baseline characteristics (Seattle Heart Failure Score, IMPACT score, and high urgent status) from their matching [39]; Rieder et al. [52] did not consider the RESP score and PRESERVE score in the matching). Overall, the funnel plot shows no evidence of publication bias in PSM studies (Additional file 1: Fig. S7). The randomized controlled trials raised a few concerns as well. In general, the assessment of risk of bias was performed very strictly. Studies that did not follow the intention-to-treat protocol were rated as "serious risk". Likewise, studies with drop-outs were rated as "serious risk". If a parameter mentioned in the study protocol was changed or not analysed without justification, this also resulted in a rating of "serious risk". However, we are convinced that the overall quality of the RCTs is good and that there is no publication bias (Additional file 1: Fig. S8).

## Discussion

This meta-analysis comprises data of 34 studies, including a total of 1297 patients treated with CytoSorb® compared to 1314 controls, in order to evaluate the use of CytoSorb® absorbers. We did not observe a significant reduction in mortality due to CytoSorb® intervention.

The use of blood purification methods in the treatment of severely-ill patients suffering from infection, sepsis and multiple organ failure has been investigated for over 30 years. [59] In a cytokine storm the massive release of cytokines can cause a severe inflammatory syndrome that leads to organ dysfunction [60]. Thus, the removal of pro-inflammatory cytokines such as IL-1β, IL-6, IL-8 and TNF-α from the blood stream should curb the hyperactivation of the immune system and reduce the systemic response. From this hypothesis, varying justifications for hemoadsorption arise. The removal of a single targeted cytokine was unsuccessful in the past, so that efforts were concentrated on the unselective removal of cytokines via blood purification methods such as by hemoperfusion using adsorber systems such as CytoSorb® [61, 62]. The use of CytoSorb® and other adsorbers is widespread, although to-date there is no consensus on the effectiveness of treatment. [63, 64]

One limitation of our meta-analysis is the heterogeneity of the studies. The observations of individual studies differed substantially, which we attribute to various reasons. First, there are a fair number of studies with only few study participants yet large effect sizes. Eight studies included less than 15 patients per group [26, 28, 39, 45, 46, 51, 56, 58]. These studies achieved great effects with CytoSorb® intervention. By contrast, there are larger RCTs and observational studies with propensity score matching (PSM) with notably smaller effect sizes. Secondly, the use of CytoSorb® differed in many ways. The number of adsorbers, duration of therapy, time from diagnosis to first use of the adsorber, and blood flow rate were very inconsistent. Thirdly, medical conditions were compared that differ completely in their pathophysiology. For this reason, we included the subgroup analysis of different medical conditions. Finally, the variances proved to be quite large due to the non-normal distribution of some variables. We overcame this limitation by performing subgroup analyses. We found no positive effect of CytoSorb® in any of the examined subgroups. This is true for the subgroups of different medical conditions as well as for the subgroup of different types of study design.

Not only is the positive effect of CytoSorb® intervention in terms of mortality reduction uncertain, even studies investigating the theoretical rationale behind treatment, namely the reduction in blood cytokine levels, show divergent results. The CytoSorb® adsorber has been shown to remove IL-1β, IL-6, IL-8, and TNF α in vivo, ex vivo and in vitro studies [65, 66]. Other studies have shown no significant reduction in IL-6 levels [32, 50]. A possible explanation provided by Honore et al. for stable cytokine levels despite removal during hemoabsorption is the shift of further cytokines from the interstitium into the bloodstream [67]. Furthermore cytokine release is continuous, so that a selective removal for a few hours a day may not be sufficient to have an impact on treatment success. Both are possible reasons why perhaps CytoSorb® failed to reduce mortality. Also it has been argued that some of the disease states, in which CytoSorb® is used, mainly SARS-CoV-2 and cardiopulmonary bypass surgery are not accompanied by particularly high cytokine levels [32, 50]. Assuming an adequate removal of pro-inflammatory cytokines, a further explanation for the failure to reduce mortality with Cytosorb® is the unselective removal of all hydrophobic plasma components ranging up to molecular mass of 55 kDa. This means that anti-inflammatory mediators, hormones and clotting factors are also removed [68]. In addition, antibiotics are also removed by the Cytosorb® adsorber, which may cause subtherapeutic antibiotic levels, impacting the success of sepsis treatment and overall survival [69].

We urgently need evidence from randomized controlled trials for the various medical conditions in which CytoSorb® is used taking into account the dynamics of the different diseases. At the moment only the use in CPB might be sufficiently assessable as shown by the trial sequential analysis.

## Conclusion

In conclusion, there is no evidence of a reduction in mortality by treatment with CytoSorb® in any of the examined conditions. Therefore, we cannot recommend the use of CytoSorb® in intensive care patients unless clear evidence is generated. We need adequately designed RCTs in specific medical conditions targeting the right patients. Hence, ground work needs to be undertaken to identify patients likely to respond to the therapy, e.g. very high cytokine levels, and the optimal time point for therapy of the respective condition.

## Supplementary Information

**Additional file 1. Fig. S1:** 30-day mortality by different diagnoses. **Fig. S2:** In-hospital mortality by different diagnoses. **Fig. S3:** ICU mortality by different diagnoses. **Fig. S4:** Risk of Bias in Non-randomized Studies of Interventions. **Fig. S5:** Risk of Bias in randomized trials. **Fig. S6:** Funnel plot for nPSM. **Fig. S7:** Funnel plot for PSM. **Fig. S8:** Funnel plot for RCT. **Table S1:** Baseline SOFA, SAPS-2, APACHE-2 and EuroScore-2 (difference: treatment group – control group). **Table S2:** CRP and PCT before and first reported after treatment (difference: treatment group – control group). **Table S3:** Lactate and IL-6 before and first reported after treatment (difference: treatment group – control group).

## Data Availability

As the meta-analysis is based on published data only, all data are publicly available.
