# Supplementary material for: Efficacy of CytoSorb®: a systematic review and meta-analysis
Source: Crit Care. 2023 May 31;27:215. doi: 10.1186/s13054-023-04492-9 (PMC10230475; doi:10.1186/s13054-023-04492-9)
Supplement: Supplementary file 1 — Additional file 1. Fig. S1: 30-day mortality by different diagnoses. Fig. S2: In-hospital mortality by different diagnoses. Fig. S3: ICU mortality by different diagnoses. Fig. S4: Risk of Bias in Non-randomized Studies of Interventions. Fig. S5: Risk of Bias in randomized trials. Fig. S6: Funnel plot for nPSM. Fig. S7: Funnel plot for PSM. Fig. S8: Funnel plot for RCT. Table S1: Baseline SOFA, SAPS-2, APACHE-2 and EuroScore-2 (difference: treatment group – control group). Table S2: CRP and PCT before and first reported after treatment (difference: treatment group – control group). Table S3: Lactate and IL-6 before and first reported after treatment (difference: treatment group – control group). [file 13054_2023_4492_MOESM1_ESM.docx]

**Supplement Figures and Tables:**

**Supplement Fig. 1: 30-day mortality by different diagnoses**


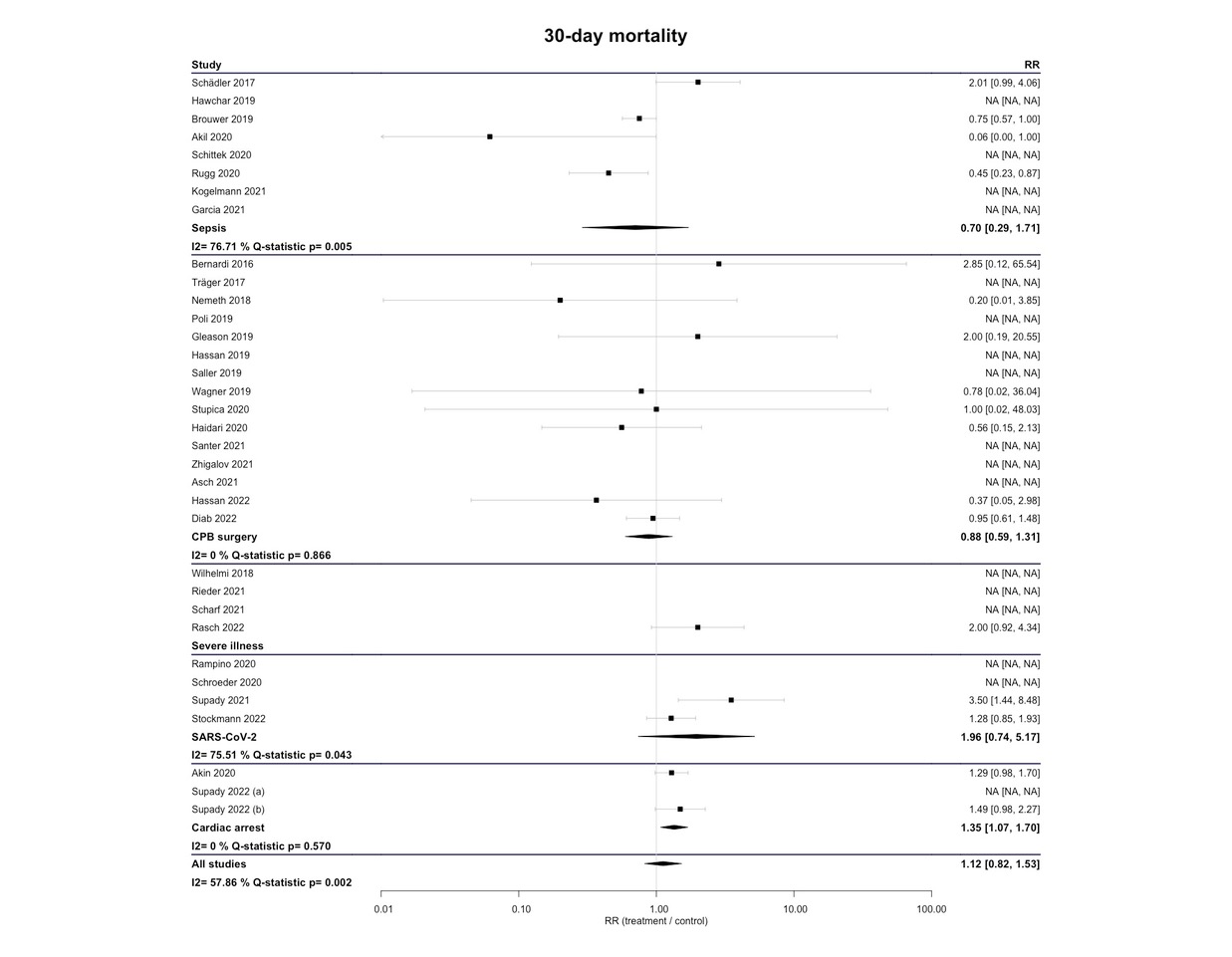


**Supplement Fig. 2: In-hospital mortality by different diagnoses**

**
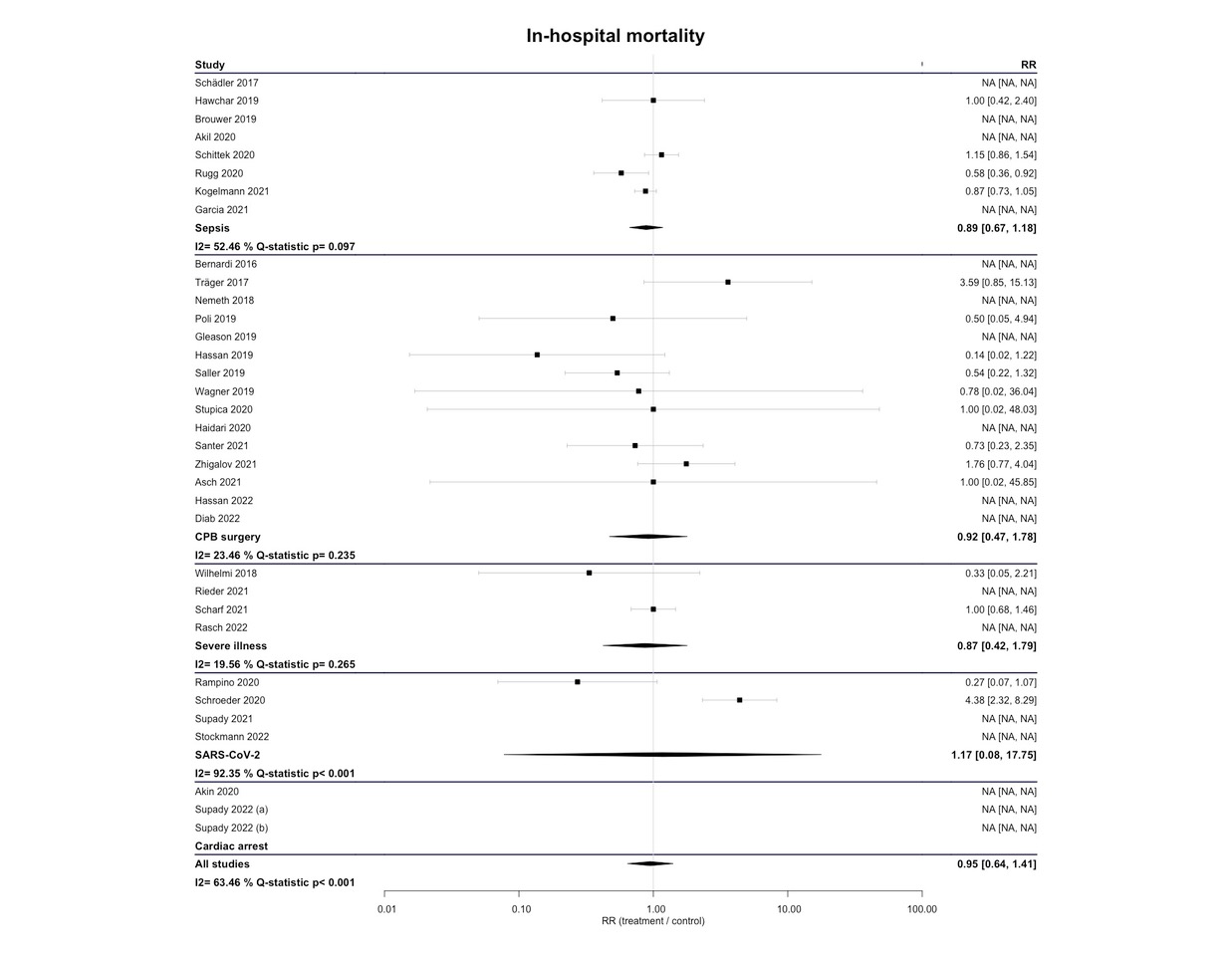
**

**Supplement Fig. 3: ICU mortality by different diagnoses**

**
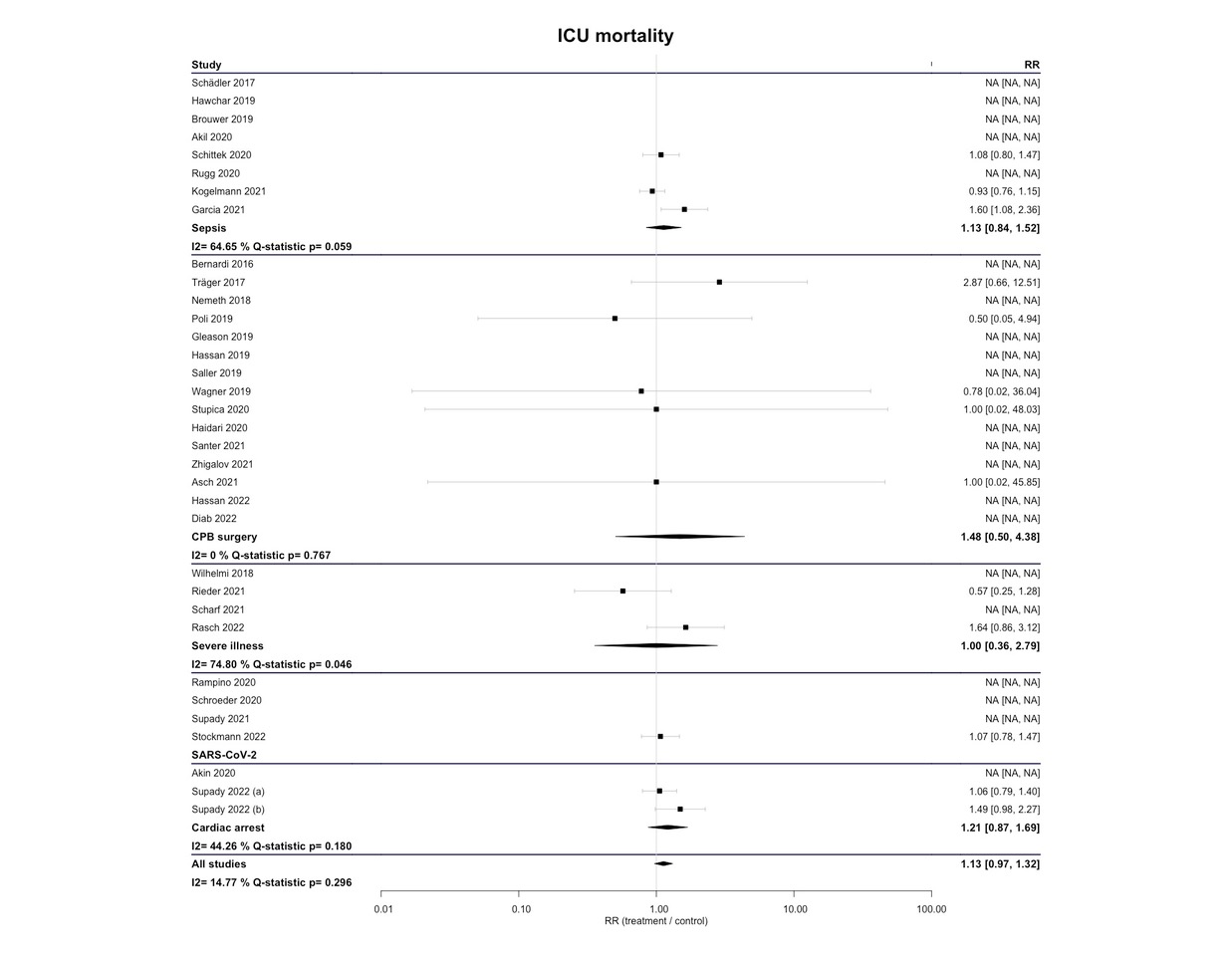
**

**Supplement Fig. 4: Risk of Bias in Non-randomized Studies of Interventions**

**
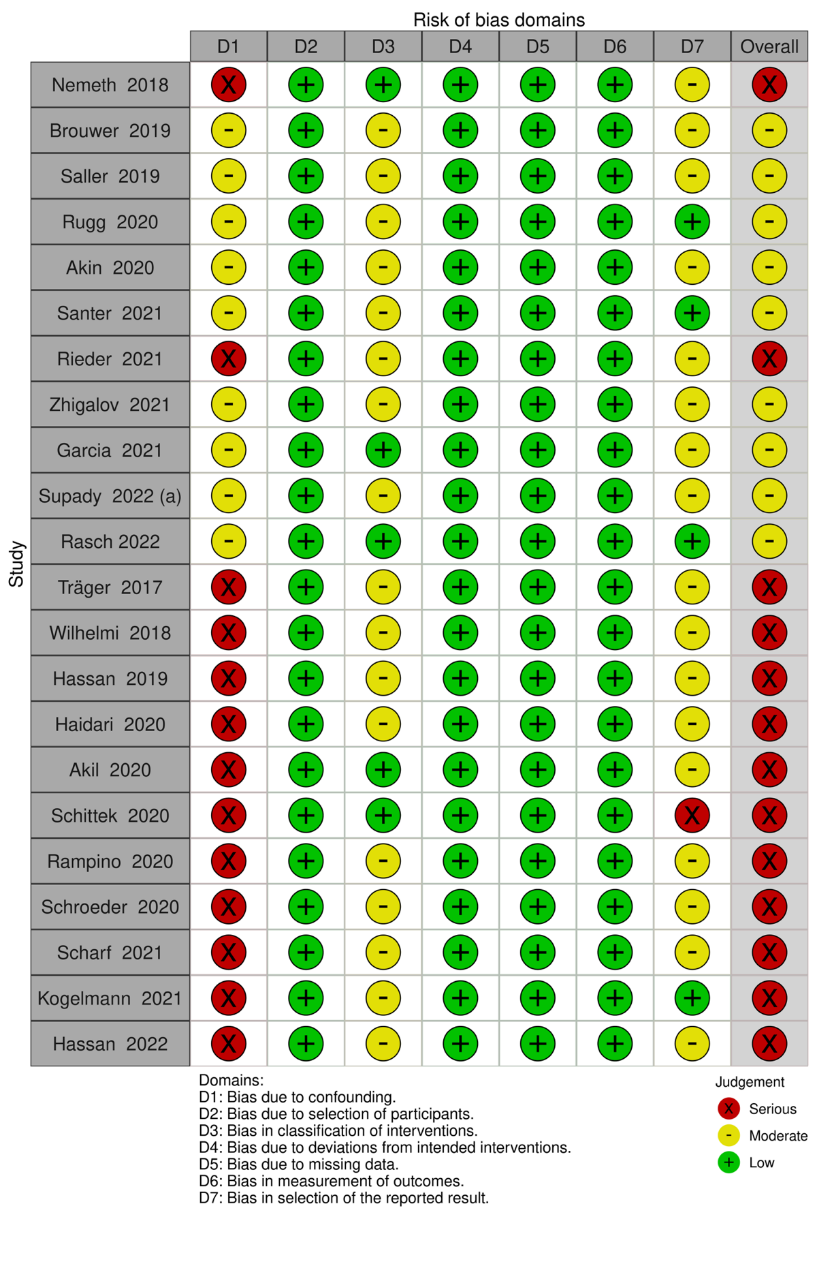
**

**Supplement Fig. 5: Risk of Bias in randomized trials**

**
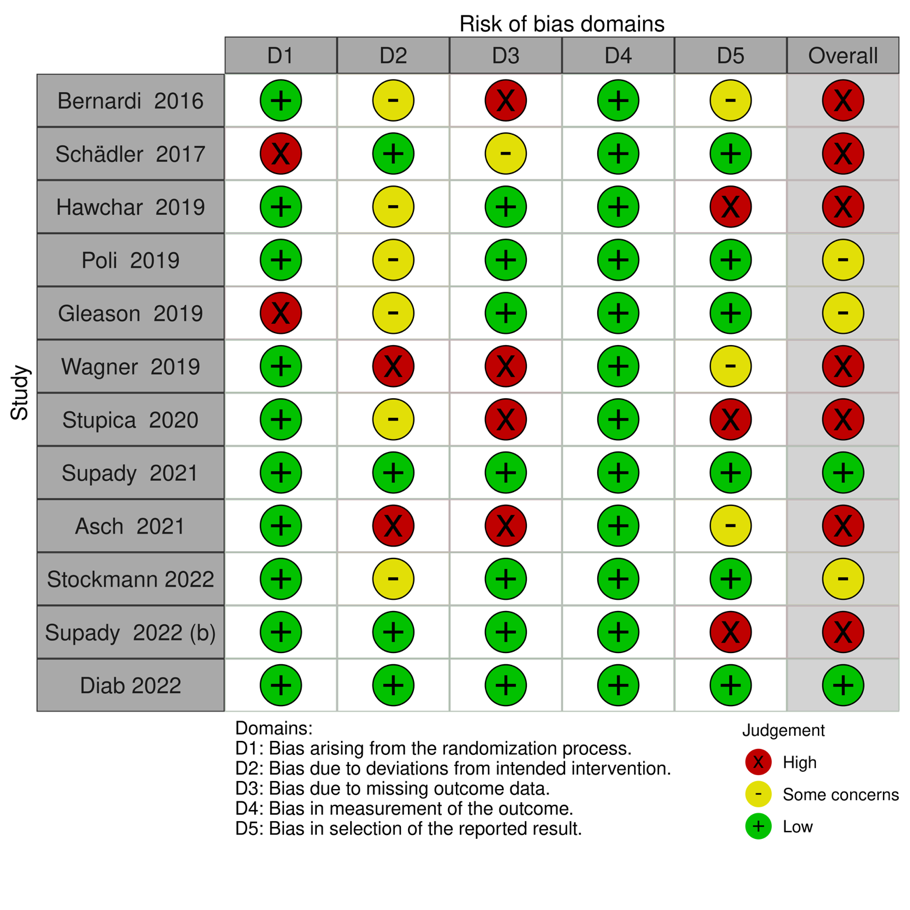
**

**Supplement Fig. 6: Funnel plot for nPSM**

**
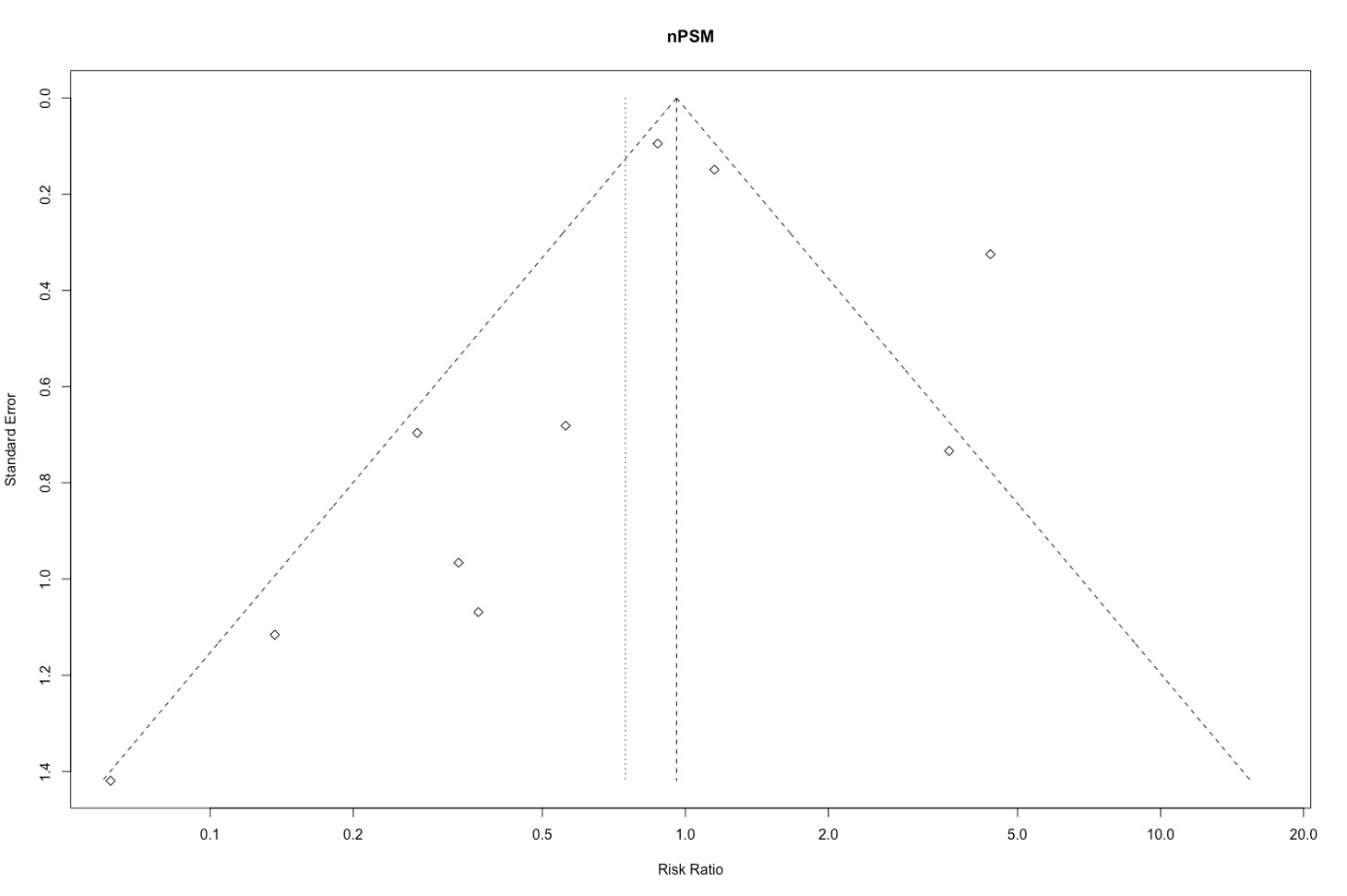
**

nPSM non propensity score matched cohort studies

**Supplement Fig. 7: Funnel plot for PSM**

**
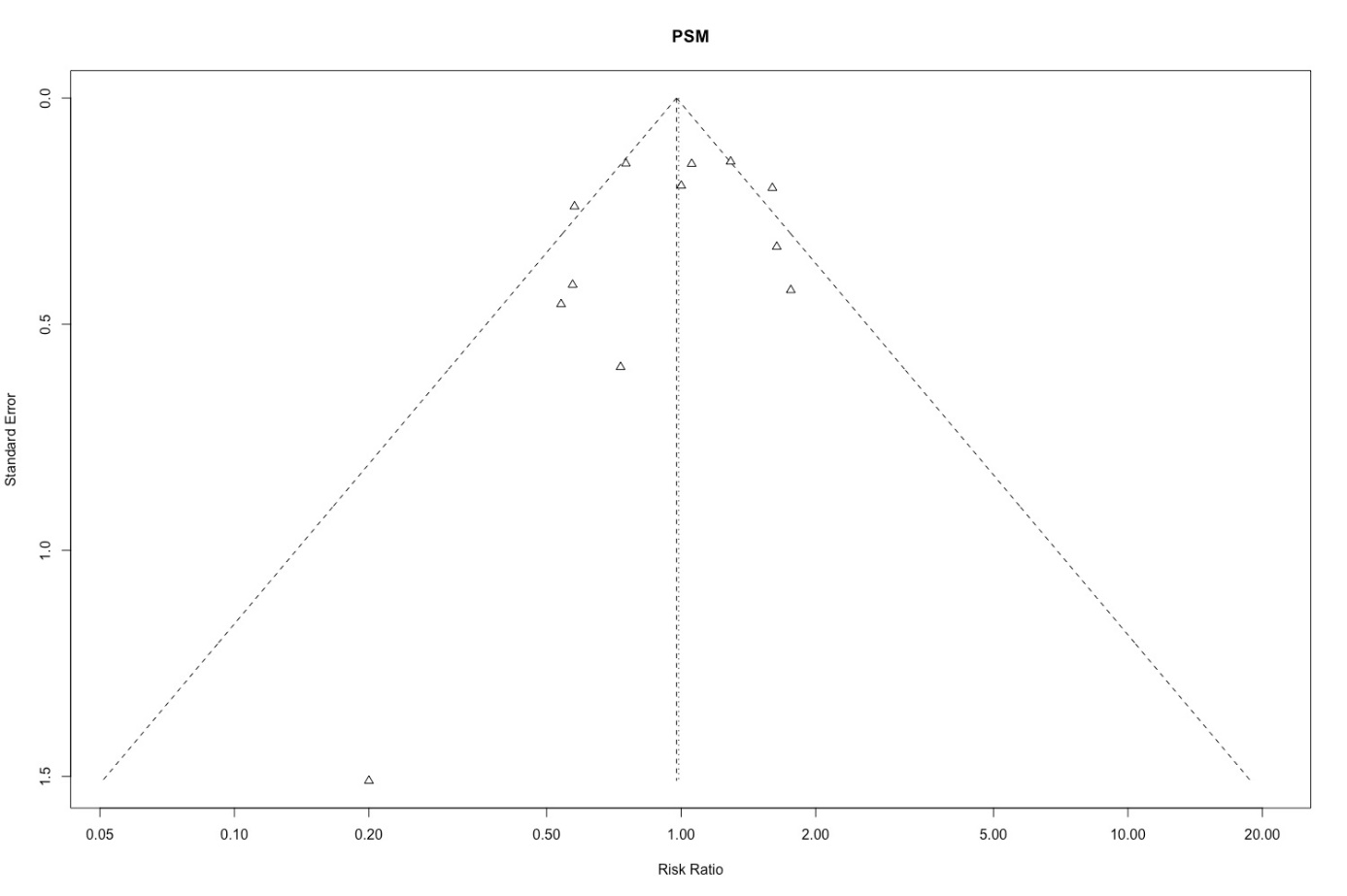
**

PSM propensity score matched cohort studies

**Supplement Fig. 8: Funnel plot for RCT**

**
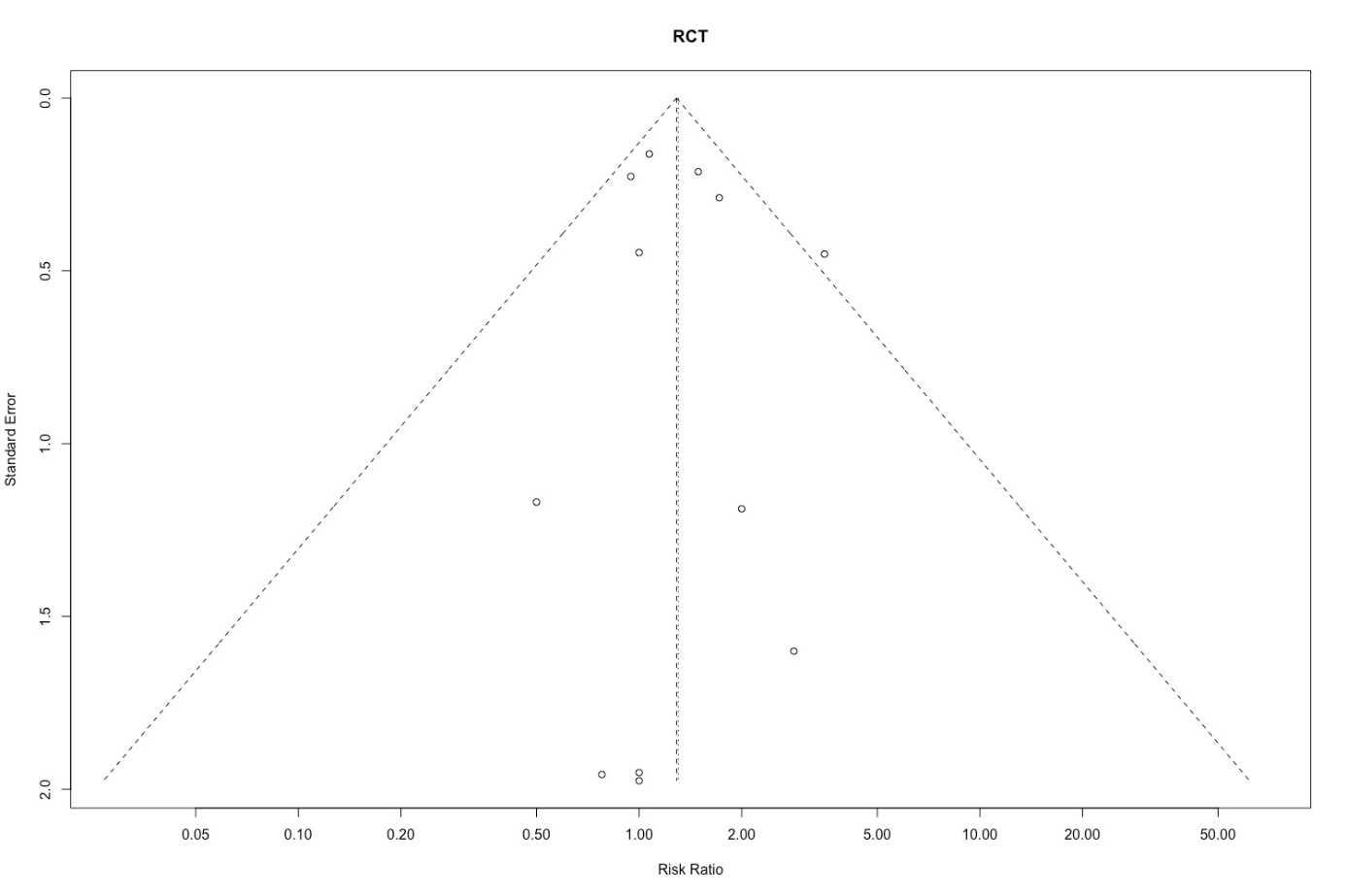
**

RCT randomized controlled trial

**Supplement Tab. 1: Baseline SOFA, SAPS-2, APACHE-2 and EuroScore-2 (difference: treatment group – control group)**

|  | **SOFA** | | **SAPS-2** | | **APACHE-2** | |  | |
| --- | --- | --- | --- | --- | --- | --- | --- | --- |
|  | **M±SD** | **Md [IQR]** | **M±SD** | **Md [IQR]** | **M±SD** | **Md [IQR]** |  |  |
| **Sepsis** | 0.06 (± 1.36) | 0.33 [-0.59; 1.24] | -1.44 (± 7.93) | -0.78 [-7.33; 5.78] | -2.84 (± 3.69) | -3.15 [-6.79; 0.49] |  |  |
| **Severe illness** |  |  |  |  |  |  |  |  |
| **SARS-CoV-2** |  |  |  |  |  |  |  |  |
| **Cardiac arrest** | -0.15 (± 1) | -0.20 [-1.25; 0.84] | 1.22 (± 4.1) | 1.12 [-3.26; 5.49] |  |  |  |  |
| **All studies** | -0.18 (± 0.72) | 0.09 [-0.50; 0.68] | -0.65 (± 4.35) | 0.20 [-3.14; 3.54] | -1.73 (± 2.54) | -1.89 [-4.68; 0.90] |  |  |

**Supplement Tab. 2: CRP and PCT before and first reported after treatment (difference: treatment group – control group)**

|  | **CRP (mg/l) before treatment** | | **CRP (mg/l) after treatment** | | **PCT (ng/ml) before treatment** | | **PCT (ng/ml) after treatment** | |
| --- | --- | --- | --- | --- | --- | --- | --- | --- |
|  | **M±SD** | **Md [IQR]** | **M±SD** | **Md [IQR]** | **M±SD** | **Md [IQR]** | **M±SD** | **Md [IQR]** |
| **Sepsis** | 1.81 (± 35.3) | -1.53 [-34.62, 31.56] | 3.14 (± 76.74) | 10.21 [-77.92; 98.33] | -7.60 (±7.76) | -7.13 [-13.29; -0.97] | 5.09 (± 9.9) | 5.69 [-3.42; 14.80] |
| **Severe illness** |  |  | 46.49 (±49.19) | 43.76 [-13.89; 101.41] |  |  |  |  |
| **SARS-CoV-2** |  |  | -5.97 (± 43.51) | -5.37 [-53.95; 43.20] | 2.06 (± 1.88) | 0.61 [-1.09; 2.30] |  |  |
| **Cardiac arrest** |  |  | -92.14 (±245.76) | -95.08 [-324.10; 133.94] | -1.02 (± 2.82) | -1.03 [-3.83; 1.78] |  |  |
| **All studies** | 6.89 (±28.01) | -0.30 [-31.21; 31.80] | 9.08 (± 31,63) | 7.26 [-28.30; 42.83] | -0.49 (± 2.07) | -0.46 [-1.91; 0.99] | 4.42 (± 7.12) | 1.36 [-2.27; 5.00] |

Only the mean differences shown in red was significant at the 0.05 level. Otherwise no statistically significant differences occurred.

**Supplement Tab. 3: Lactate and IL-6 before and first reported after treatment (difference: treatment group – control group)**

|  | **Lactate (mmol/l) before CytoSorb** | | **Lactate (mmol/l) after CytoSorb** | | **IL-6 before CytoSorb** | | **IL-6 after CytoSorb** | |
| --- | --- | --- | --- | --- | --- | --- | --- | --- |
|  | **M±SD** | **Md [IQR]** | **M±SD** | **Md [IQR]** | **M±SD** | **Md [IQR]** | **M±SD** | **Md [IQR]** |
| **Sepsis** | 0.88 (±2.15) | 0.99 [-0.20; 2.18] | 0.29 (± 0.79) | 0.29 [-0.50; 1.09] | 443 (± 520) | -5962.57 [-20364.73; 8439.59] | -100 (±235) | -158.20 [-394.16; 77.76] |
| **Severe illness** | 2.52 (± 2.93) | 2.36 [-1.08; 5.79] | -0.76 (±2.06) | -0.83 [-3.21; 1.54] | 19818 (±48381) | 5055.28 [1620.73; 8489.84] |  |  |
| **SARS-CoV-2** |  |  |  |  | -104 (±427) | -58.57 [-467.34; 350.21] |  |  |
| **Cardiac arrest** | -0.31 (± 1.81) | -0.67 [-3.09; 1.74] | -1.08 (± 1.44) | -0.68 [-2.23; 0.87] | -226 (± 327) | -275.00 [-620.44; 70.44] |  |  |
| **All studies** | 0.60 (± 0.91) | 0.60 [-0.32; 1.53] | 0.08 (± 0.30) | 0.06 [-0.26; 0.38] | 121.31 (±526.13) | -19.89 [-500.92; 461.13] | -17,76 (±158.4) | 12.26 [-112.44; 136.96] |

Only the mean differences shown in red was significant at the 0.05 level. Otherwise no statistically significant differences occurred.
